# Phylogenomics of the rarest animals: a second species of Micrognathozoa identified by machine learning

**DOI:** 10.1098/rspb.2024.2867

**Published:** 2025-02-19

**Authors:** Shoyo Sato, Cecilie Appeldorff, Owen S. Wangensteen, Sandra Garcés-Pastor, Christopher E. Laumer, María Herranz, Gonzalo Giribet, David Renault, Peter Rask Møller, Katrine Worsaae

**Affiliations:** ^1^ Marine Biological Section, Department of Biology, University of Copenhagen, Copenhagen 2100, Denmark; ^2^ Department of Evolutionary Biology Ecology and Environmental Sciences & Biodiversity Research Institute (IRBio), Universitat de Barcelona, Barcelona, Catalonia 08028, Spain; ^3^ Institute of Marine Sciences (ICM), CSIC, Barcelona, Catalonia 08003, Spain; ^4^ Department of Life Sciences, The Natural History Museum, London, UK; ^5^ Area of Biodiversity and Conservation, Superior School of Experimental Science and Technology (ESCET), Rey Juan Carlos University, Móstoles, Madrid, Spain; ^6^ Global Change Research Institute (IICG-URJC), Móstoles, Madrid 28933, Spain; ^7^ Museum of Comparative Zoology, Department of Organismic and Evolutionary Biology, Harvard University, Cambridge, MA, USA; ^8^ UMR CNRS 6553 ECOBIO (Ecosystèmes, biodiversité, évolution), Université Rennes, avenue du Général Leclerc, Rennes cedex 35042, France; ^9^ Institut Universitaire de France, Paris, France; ^10^ Natural History Museum of Denmark, University of Copenhagen, Copenhagen 2100, Denmark

**Keywords:** meiobenthos, Spiralia, phylotranscriptomics, species delimitation, confocal laser scanning microscopy, integrated taxonomy

## Abstract

The latest animal phylum to be discovered, Micrognathozoa, constitutes a rare group of limnic meiofauna. These microscopic ‘jaw animals’ are among the smallest metazoans yet possess highly complex jaw structures. The single species of Micrognathozoa, *Limnognathia maerski* Kristensen and Funch, 2000, was first described from Greenland, later reported from a remote Subantarctic island and more recently discovered in the Pyrenees on the European continent. Successful collections of these three known populations facilitated investigations of the intraphylum relationships and species limits within *Limnognathia* for the first time. Through detailed anatomical comparisons, we substantiate the lack of morphological differences between the three geographically disjunct populations. With transcriptomic data from single specimens, we conducted the first intraphylum phylogenetic analyses and extensively tested species hypotheses using standard approaches and novel machine learning methods. Analyses clearly delimited the Subantarctic population, here described as *Limnognathia desmeti* sp. nov., the second species of Micrognathozoa, but did not definitively split the Greenland and Pyrenees populations as separate species. Divergence dating analysis suggests the disjunct distribution of Micrognathozoa is not human mediated but the result of long-distance dispersal raising questions about their dispersal capabilities and potential undiscovered populations.

## 1. Background

**Figure 1.**
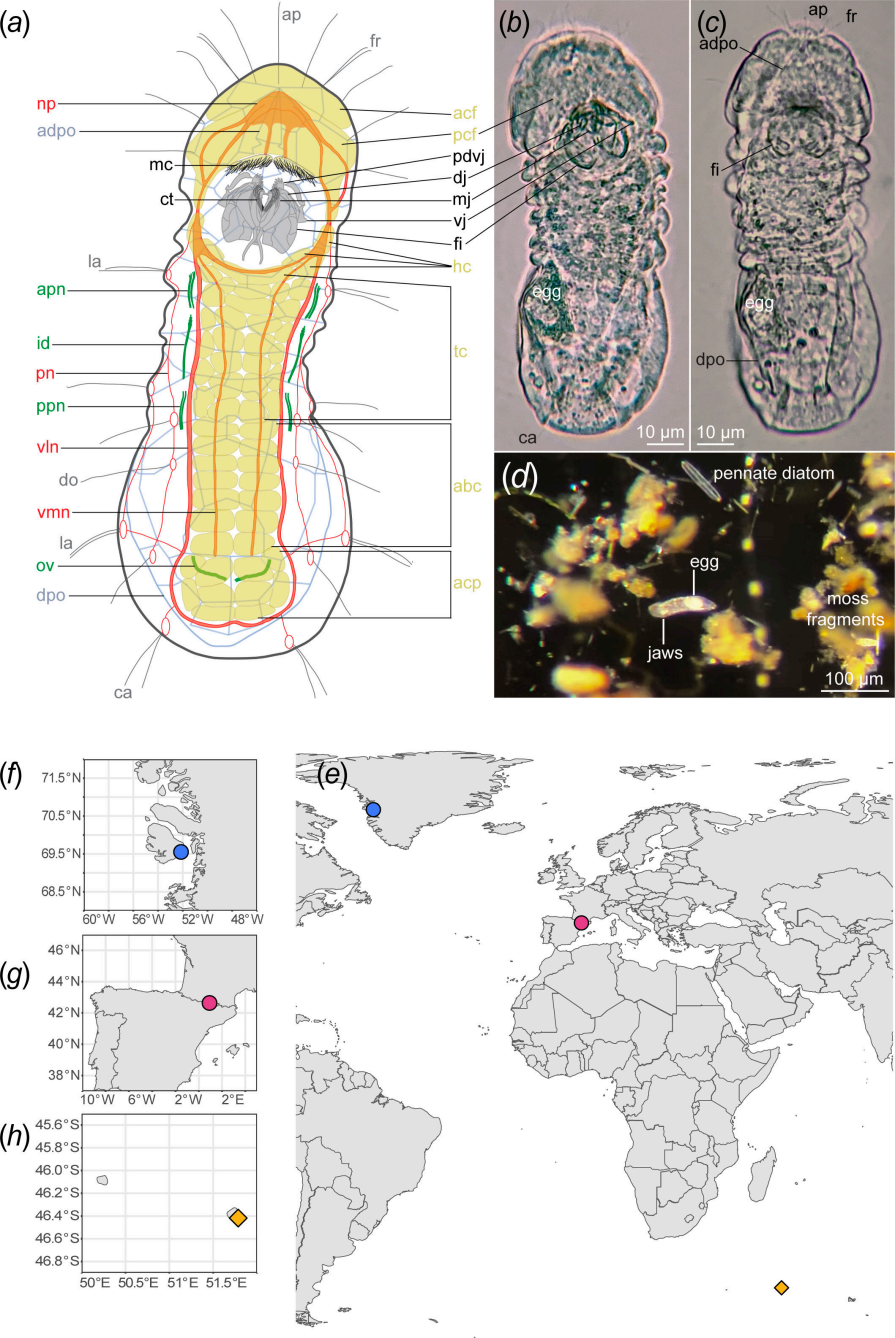
Distribution and habitus of Micrognathozoa. (*a*) Schematic of Micrognathozoa showing key morphological characteristics investigated in this study. (*b*) Ventral and (*c*) LM of *Limnognathia desmeti* sp. nov. (paratype, NHMD-001801226). (*d*) Dissecting scope image of *Limnognathia desmeti* sp. nov. among sampled substrate. (*e*) Map of the three populations of Micrognathozoa sampled for this study. Circles denote populations of *Limnognathia maerski* and diamonds denote *Limnognathia desmeti* sp. nov. (*f*) Isunngua Spring, Disko Island, Greenland. (*g*) Bassa Nera, Aigüestortes i Estany de Sant Maurici NP, Catalunya. (*h*) Pointe du Bougainville, Île de la Possession, Crozet Archipelago. Colours in the map correspond to the colours used in all other figures. abc, abdominal ciliophores; acf, anterior ciliated field; acp, adhesive ciliary pad; adpo, apical dorsal plate outline; ap, apicalia; apn, anterior protonephridia; ca, caudalia; ct, ciliary tufts; dj, dorsal jaw; do, dorsalia; dpo, dorsal plate outline; egg, egg; fi, fibularium; hc, head ciliophores; id, intermediate duct; la, lateralia; mc, mouth ciliation; mj, main jaw; np, neuropil; ov, oviduct; pcf, posterior ciliated field; pdvj, pseudodigit of the ventral jaw; pn, peripheral nerve; ppn, posterior protonephridia; tc, trunk ciliophores; vln, ventro-lateral nerve; vj, ventral jaw; vmn, ventro-median nerve.

Micrognathozoa (figure 1*a*) was first described in 2000 from a single species, *Limnognathia maerski* Kristensen and Funch, 2000, from cold springs on Disko Island, Greenland [1]. An affinity with Gnathostomulida and particularly Syndermata (syn. Rotifera *sensu lato*), a grouping called Gnathifera, was soon hypothesized based on morphological similarities [1–6]. Molecular phylogenetic analyses testing the position of Micrognathozoa found that it did not nest within known phyla [7,8] and Micrognathozoa was elevated in status to become the most recently described and only exclusively freshwater animal phylum. Phylogenomic analyses of Spiralia later firmly placed Micrognathozoa as sister phylum to Syndermata [9–11], together called Gynognathifera [12], within the larger clade of Gnathifera. Despite their minuscule size with a maximum length of 150 µm, Micrognathozoa possess the most complex jaw structures in the animal kingdom [1–3]. No males of the species have been found and the phylum has been proposed to reproduce primarily or solely by parthenogenesis [1,8,13].

Soon after the description of the Greenlandic species, Micrognathozoa were reported from Île de la Possession, in the Subantarctic Crozet Archipelago (hereon Crozet) [2]. Despite their distance from Greenland, because of their seemingly identical morphology, the Crozet specimens were also assigned to *L. maerski* [2]. A few additional specimens were later reported from Wales and Berkshire, UK [13], and sequence matches in environmental DNA (eDNA) metabarcoding samples indicated micrognathozoan presence in continental Europe [14,15]. However, a European population was never localized or collected until the recent discovery of Micrognathozoa in the Pyrenees [16]. Currently, a single species is described in the phylum Micrognathozoa. Only data from pooled Greenlandic specimens have been included in molecular phylogenetic analysis (e.g. [7,10,11,17]). Neither the intraphylum relationships among populations nor potential cryptic speciation have been investigated. Through extensive sampling efforts over the last 10 years, we obtained multiple individuals from each of the three known populations of *Limnognathia*: Greenland, Pyrenees and Crozet (figure 1*e*–*h*). Those samples are here used in the first phylogenetic analysis of the internal relationships within Micrognathozoa using phylotranscriptomics. The hypothesis of cryptic speciation is tested with advanced microscopy and novel machine learning species delimitation analyses.

We approach integrative species delimitation from species discovery to validation using a range of methods and data types [18]. Species discovery methods, agnostic to species hypotheses, do not require *a priori* labelling of individuals to species [18]. Morphological species discovery is nearly impossible in cryptic, morphologically conserved groups like soft-bodied meiofauna [19,20], resulting in underestimates of true diversity. Common molecular species delimitation methods oversplit and identify highly divergent populations as species, particularly in systems that do not meet model assumptions, lack informed priors and have limited dispersal [21–24].

Unsupervised machine learning (UML) clustering methods are agnostic to population parameters and species divergences. These parameters are difficult to estimate in meiofauna where baseline knowledge is poor compared with well-studied groups. UML methods were not specifically developed for the problem of species delimitation but can reveal underlying structures in the data that could correspond to species [25,26].

Species validation methods explicitly test *a priori* species hypotheses [18]. Common methods that rely on the multi-species coalescent are limited to divergence-only models, which might not reflect the underlying speciation processes [27]. Supervised machine learning (SML) has allowed testing of models using broader assumptions and non-divergence processes [27], and has even inferred unknown species boundaries using knowledge from biologically similar systems [28–30].

Standard integrative methods can produce contradictory results by both over- and under-splitting species numbers [28]. Due to its conserved morphology, high habitat specificity, geographic isolation, lack of precise priors and putative parthenogenesis, Micrognathozoa is an ideal case to test the novel methods of both agnostic (UML) and informed (SML) machine learning.

## 2. Methods

Detailed methodology can be found in the electronic supplementary material.

### (a) Specimen collection

Specimens were collected by examining water squeezed and filtered from aquatic moss samples under a dissecting scope and isolating them with pipettes. Specimens were anaesthetized with MgCl_2_ and fixed for morphological study or stored in RNA*later* or flash frozen for molecular study.

### (b) Morphological study

Light microscopy (LM) images and videos were obtained using a LabCam (iDu optics) mounted iPhone 11. Immunohistochemistry and confocal laser scanning microscopy (CLSM) followed previously described methodology [6] (electronic supplementary material, tables S1 and S2) using an Olympus FV-1000. Z-stacks were analysed with Imaris (Oxford Instrument 2022). Comparisons of the jaws are based on previously published scanning electron microscopy (SEM) images [2,3].

### (c) Sequencing

Single-individual transcriptome libraries were generated using the Clonetech SMART-Seq HT and Nextera XT DNA kits. Sequencing and demultiplexing was done on an Illumina NovaSeq 6000. Outgroup sequences were obtained from NCBI (electronic supplementary material, table S3 for taxon sampling). A 684 bp reference Sanger cytochrome *c* oxidase subunit I (COI) was sequenced from the cDNA using universal primers (electronic supplementary material, table S4) [31], purified with the E.Z.N.A. Cycle Pure Kit, sequenced at Macrogen Europe and assembled in Geneious [32].

### (d) Transcriptome assembly and orthology inference

All transcriptomes (electronic supplementary material, table S3) were assembled and processed following published pipelines [33–39]. Assembly quality was assessed with BUSCO and the Metazoa Orthodb v10 [40,41]. Orthology inference of all transcriptomes followed the PhyloPyPruner (https://pypi.org/project/phylopypruner/) pipeline [42–48] to create 50 and 70% occupancy matrices (matrices M1 and M2, respectively).

A 100% occupancy single-nucleotide polymorphism (SNP) (matrix M3) and 18S rRNA datasets were created following GATK ‘best practices’ [49–52]. Unlinked SNP datasets were created with randSNPs_from_vcf.pl. Resulting VCF files were filtered at 50% occupancy (matrix M4) and 100% occupancy (matrices M5–M9), converted to phylip with vcf2phylip.py [53] and processed using phrynomics [54]. COI sequences were extracted with MitoGeneExtractor [55] using the generated Sanger reference and aligned with MAFFT [44]. Poorly aligned bases were trimmed (alignment = 612 bp).

A 100% occupancy matrix of Metazoa_odb v10 BUSCO loci (matrix M10) was extracted from *Limnognathia* assemblies with TOAST [56], aligned with MAFFT and processed with Gblocks [57,58] to obtain single-copy nucleotide orthologues. Information of all matrices used in this study can be found in electronic supplementary material, table S5.

### (e) Phylogenetic analyses

Maximum likelihood phylogenetic analyses were conducted in IQ-TREE [59–63]. Bayesian analyses were run in ExaBayes [64]. Log files were combined with LogCombiner [65] and convergence was checked in Tracer [66].

Divergence dating analysis was conducted in BEAST [67] using the trimmed COI and 18S rRNA alignments. Substitution models were determined with ModelFinder in IQ-TREE. Universal COI (1.76%) [68] and 18S rRNA (0.02%) [69] substitution rates were used. Convergence was checked in Tracer. A maximum clade credibility tree was produced with TreeAnnotator [67].

### (f) Species delimitation

PTP and bPTP [70] were run using the maximum likelihood tree of matrix M1 as input. GMYC [71] was run using the ultrametric BEAST tree from divergence time estimation.

Data in matrix M4 were clustered with principal component analysis (PCA), random forest (RF) and t-distributed stochastic neighbour embedding (tSNE) [25,72–81]. Variational autoencoder (VAE) analysis [82] was run using matrices M3 and M5–M9. Phylip files were one-hot encoded using a custom python script OneHotEncode.py.

Matrix M4 was analysed with SNAPPER [83]. The Yule model lambda parameter was estimated with Pyule [84]. Two xml files were created with BEAUti [67] corresponding to two and three species models. Marginal likelihood estimates were calculated with PathSampleAnalyser, and models were selected with Bayes factor (BF).

DelimitR [27] was run using an MSFS file that was created from matrix M4 with easySFS [85]. CLADES analysis [29] followed Derkarabetian *et al*. [28] with matrix M10 using the ‘general’ model provided by Pei *et al*. [29] and a custom model created from a rotifer transcriptome dataset (electronic supplementary material, figure S1 and Table S6) using Libsvm [86]. Admixture analysis [87] was conducted on matrix M4.

### (g) Molecular species description

Pairwise COI distances between all specimens were computed in MEGA [88] under the K2P model [89]. The 18S rRNA and COI alignments were visualized in Geneious [32] to identify fixed molecular differences between species.

## 3. Results and discussion

### (a) Morphological observations and comparison

We found no significant differences using advanced microscopic techniques between Northern Hemisphere (Greenland and Pyrenees) and Southern Hemisphere (Crozet) individuals (electronic supplementary material, table S7).

Specimens from all populations did not differ in total body length and had three main body divisions (figure 1*a*–*d*). No males were found in any of the explored localities. The three previous detailed studies on the complex jaw morphology (figure 1*a*–*c*) revealed no differences between Greenlandic and Crozet *Limnognathia* except for a minor, non-diagnostic difference in the number of ‘teeth’ of the pseudodigits [*sensu* 1, 3] or pseuduncus [*sensu* 2] (pdvj, figure 1*a*). However, this seemingly reflects intraspecific variation or preservation differences since both four [1] and five [3] ‘teeth’ have been noted in both populations [2].

**Figure 2.**
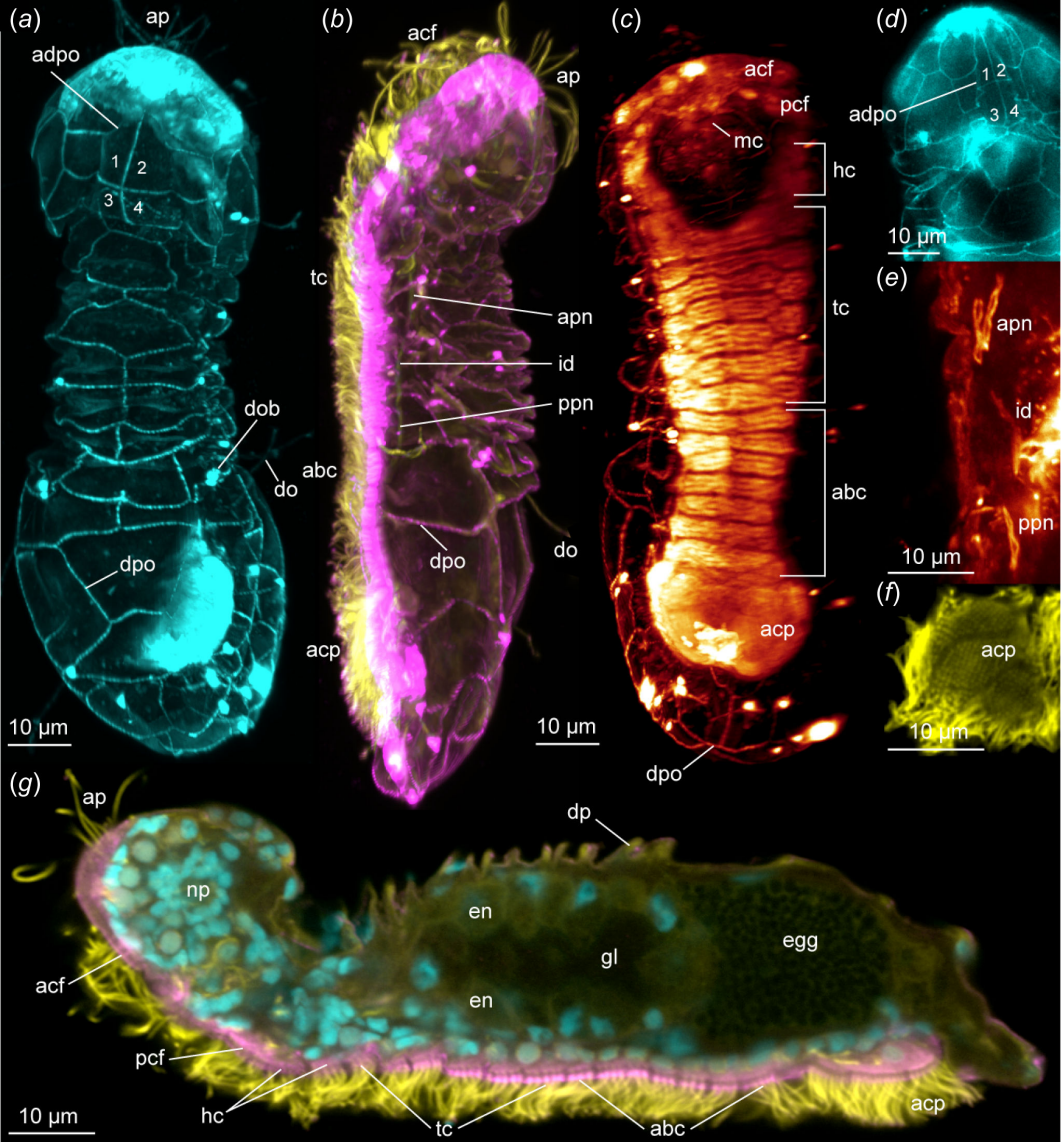
CLSM of *Limnognathia*. All images maximum intensity z-projection. Anterior end up on (*a*)–(*f*), and left on (*g*). (*a*) Dorsal *Limnognathia desmeti* sp. nov. (paratype, NHMD-001801226) whole body showing epidermal plates (cyan = anti-serotonin-like immunostaining background signal (anti-5 HT)). (*b*) Left side *Limnognathia desmeti* sp. nov. (holotype, NHMD-001801220) whole body showing cilia and epidermal plates (yellow = acetylated α-tubulin-like immunoreactivity (α-tub-LIR), magenta = anti-5 HT background signal). (*c*) Ventral *Limnognathia desmeti* sp. nov**.** (holotype) whole body showing ciliated cells (glow = α-tub-LIR). (*d*) Dorsal anterior *Limnognathia maerski* Greenland, detail of apical plate (cyan = anti-synapsin1-like immunostaining background signal (ENZO Life Sciences, ADI-VAS-SV061-E)). (*e*) Lateral thoracic section *Limnognathia desmeti* sp. nov. (paratype, NHMD-001801223) detail of nephridial structures (glow = α-tub-LIR). (*f*) Ventro-posterior abdomen of *Limnognathia desmeti* sp. nov. (paratype, NHMD-001801223) detail of adhesive ciliary pad (yellow = α-tub-LIR). (*g*) Mid-sagittal body of *Limnognathia desmeti* sp. nov. (holotype) whole body showing ciliation and internal structures (yellow = α-tub-LIR, cyan = DAPI, magenta = anti-5 HT background signal). dob, dorsalia cell body; dp, dorsal plate; en, endodermis; gl, gut lumen; all other abbreviations are identical to figure 1. Cells of the apical plate are numbered 1−4.

The dorsal and lateral epidermal cells of micrognathozoans lack a cuticle and are instead supported by an intracellular matrix (plates), first observed using LM. All dorsal plates of Greenlandic specimens were originally noted to be composed of 3−4 cells except for the single giant cell of the apical plate [1]. However, in the apical plate of Crozet specimens, De Smet distinguished four epidermal cells [2]. Later studies of Greenlandic specimens found a more exact number, shape and configuration of epidermal plates with CLSM [5,6] and that plate number seemingly equals cell number. We confirmed these results through reanalysis of these CLSM scans and further investigation of Crozet and Pyrenees specimens using immunohistochemistry and CLSM. Consistently across multiple protocols, unspecific accumulation (not staining) of background dye along cell borders and junctions allowed us to distinguish ~ 60 lateral and dorsal epidermal cells that are identical across individuals from all populations (figure 1*a*). The ‘apical plate’ always comprises four epidermal plates/cells (1–4 in figure 2*a*,*d*) with 2−4 detectable nuclei. The outside cell borders (adpo, figures 1*ac*; 2*a*,*d*) are clearly delimited by LM and immunostaining while the internal boundaries between the four cells are primarily detectable with increased intensity and magnification in CLSM (figure 2A and D).

Tubulin staining and CLSM revealed no differences in ciliation among Greenland, Pyrenees and Crozet specimens. The ventral ciliophores of Pyrenees and Crozet individuals (acf, pcf, hc, tc, abc, acp in figure 1*a*) exactly match the updated arrangement and number of Greenlandic specimens [6], which differs markedly from the original description using LM [1]. The form and number of sensory collar receptors (ap, fr, la, do, ca in figures 1*a*–*c* and 2*a*,*b,g*) corroborate previous descriptions [6] and is here clearly shown to originate between the epidermal plates (dob, figure 2*a*). The presence of the pharyngeal ciliary tuft [6] is confirmed in this study for both Greenland and Crozet specimens. Internal ciliary structures (i.e. oviducts, protonephridia) are also identical among individuals from the three populations down to the number and position of the terminal cells (figure 2e).

The nervous system of Crozet specimens, including the peripheral nerves (figure 1*a*) is identical to that described previously for Greenland specimens [6]. The presence of a pharyngeal ganglion [6], with possible homology to the buccal ganglion or group of somata in rotifers, gnathostomulids and chaetognaths [12,90], is confirmed in Crozet specimens.

Due to the consistent morphological findings across specimens from the three populations regarding jaws, ciliation, epidermis and nervous system, those traits uncovered since the original species description of *L. maerski* are here amended to the genus diagnosis.

### (b) Phylogenetics

**Figure 3.**
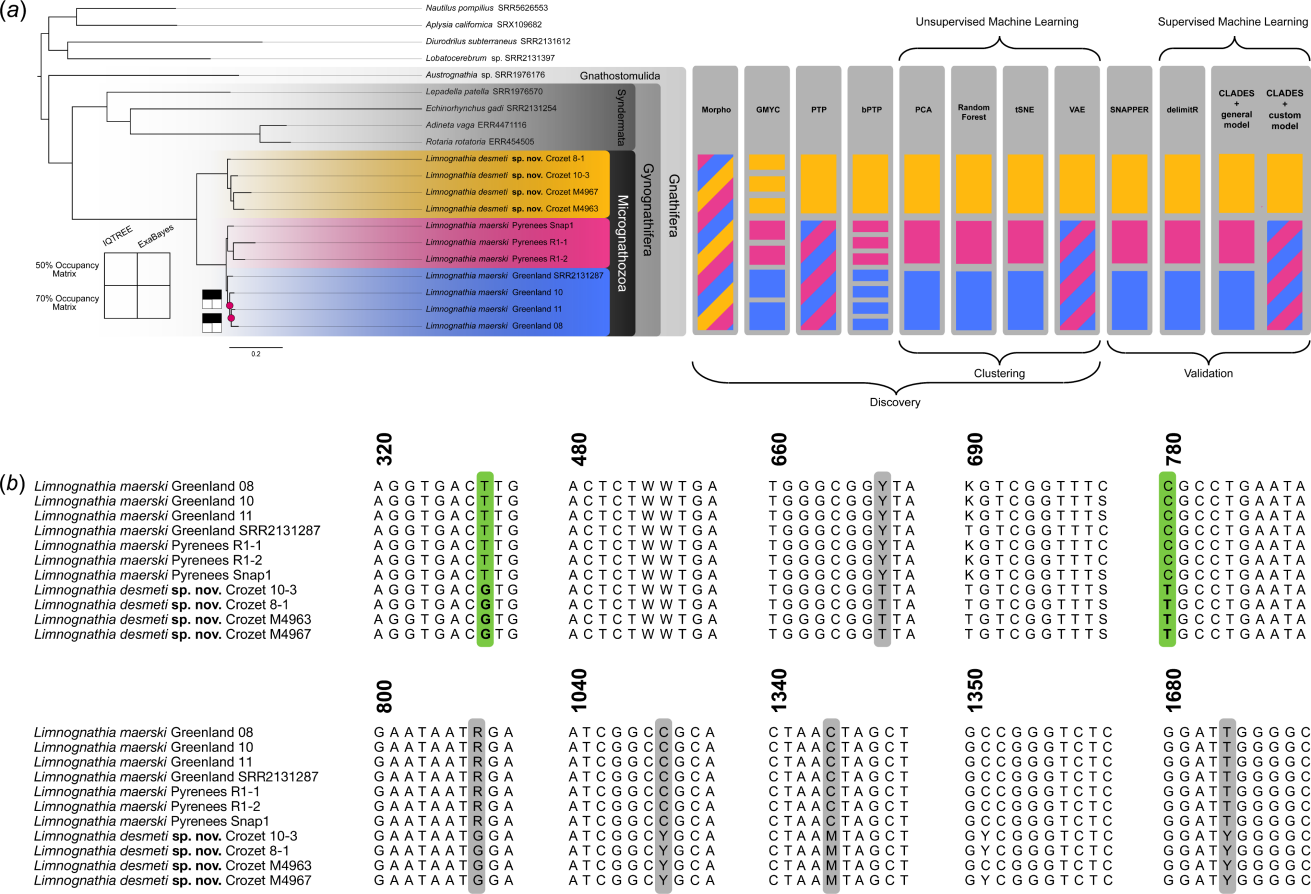
Phylotranscriptomic and species delimitation analyses of Micrognathozoa and single nucleotide polymorphisms in 18S rRNA. (*a*) Summary of phylogenetic and species delimitation analysis. Topology obtained from the maximum likelihood and Bayesian analysis of the 50% occupancy matrix. All nodes received full support in both analyses (BS = 100%, PP = 1). Differences in topology among the four analyses (50 versus 70% occupancy and maximum likelihood versus Bayesian) are summarized by the stability plots. Colours correspond to figure 1*f*–*h*. Results of species delimitation analyses are plotted to the right of the phylogeny. Blocks correspond to the number of recovered species. (*b*) Variation in 18S rRNA among *Limnognathia*. Fixed molecular diagnostic characters highlighted in green and other variable characters differing between the two species are highlighted in grey. Characters for *Limnognathia desmeti* sp. nov. are bolded.

All phylogenetic analyses recovered identical topologies except for a small difference in the relationships among Greenlandic specimens between topologies of M1 and M2 (figure 3*a*, electronic supplementary material, figures S2–S5). All nodes in all analyses had full support (bootstrap support (BS) = 100%, posterior probability (PP) = 1.00) except one node in the Greenland population of the maximum likelihood analysis of M2 (BS = 93%). We recover a monophyletic Micrognathozoa sister group to Syndermata along with monophyly of the three populations. The two Northern Hemisphere populations form a clade with a deep divergence between them and the Crozet population. The topology of all outgroup taxa reflects our current understanding of spiralian relationships (e.g. [9,10]), including Gnathifera (e.g [11]) and the nested placement of Acanthocephala within Rotifera (e.g [91,92]).

### (c) Divergence dating

We dated the split between Northern Hemisphere and Crozet populations to ~4.6 mya, 95% CI (2.38−6.65 mya) and the split between Greenland and Pyrenees populations to ~0.686 mya, 95% CI (0.29−1.09 mya) (electronic supplementary material, figure S6). Universal substitution rates were used in the absence of specific rates for Micrognathozoa or other Gnathifera. Due to their small size and soft bodies, definitive fossil calibrations are not available for this clade. Despite the caveats of using universal rates, the magnitude of the divergences is too old to be explained by human introduction but too recent to be explained by vicariance. The disjunct distribution presumably arose by long-distance dispersal, likely involving a dormant egg [1,93].

Bipolar distributions could arise by a stepping-stone dispersal scenario [94] or a one-shot jump between poles [95,96]. Regardless, many Antarctic species are allied to South America [97–99] with similar divergence time estimates to Micrognathozoa [100–102]. Floristic connectivity in the Southern Hemisphere is more closely linked to wind connectivity than geographic proximity suggesting the predominant westerlies are the main driver of dispersal in the region [103] particularly in bryophytes [104,105], including genera that harbour Micrognathozoa [99]. Micrognathozoa are difficult to find due to their size, seasonality and patchy distributions. More populations are likely to be discovered in the future possibly mediated by eDNA sequencing [15,16]. Thus, we do not rule out pole-to-pole dispersal, mountain-top hopping or wind dispersal, e.g. via South America along with their necessary substrate (i.e. mosses).

### (d) Species discovery

Molecular species discovery methods conflicted (figure 3*a*, electronic supplementary material, figures S7–S9). GMYC and bPTP both oversplit to varying degrees (seven species and eight species, respectively). bPTP results were consistent across runs, seeds and chain lengths. PTP joined the two Northern Hemisphere populations and delimited two species.

UML clustering methods were largely congruent (figure 3*a*, electronic supplementary material, figures S10–S12) with three clusters corresponding to the three populations. RF+Gap Statistic found *L. maerski* Pyrenees Snap1 in its own cluster and RF+Hierarchical Clustering found nine clusters. Hierarchical Clustering underperformed in previous analyses and recovered more clusters than other methods [25]. VAE was congruent across runs and supported two species corresponding to Northern Hemisphere and Crozet.

### (e) Species validation

Species discovery analyses mostly recovered two or three species of *Limnognathia*. Additionally, *Limnognathia* specimens from each locality likely belong to the same population because they were collected in very close proximity (e.g. from the same moss patches in a small body of water). Informed by these results, we attempted to validate either two or three species hypotheses.

SNAPPER results were consistent across step and MCMC generation configurations and preferred the three species model over the two species model (BF > 10) (figure 3*a*).

DelimitR implements RF classification by training the algorithm on data simulated under user-specified models to explicitly test species hypotheses and various population parameters including gene flow [27]. The out-of-bag error rates were small for the five models created by delimitR except those corresponding to model 3 and model 5 (electronic supplementary material, table S8). DelimitR selected the three species model without gene flow albeit with some error associated with model 5 (figure 3*a*, electronic supplementary material, figures S13*a* and Table S9). Admixture analysis confirmed the lack of gene flow among populations (electronic supplementary material, figure S13*b*).

The monogonont rotifer *Brachionus plicatilis* species complex shares many biological characteristics with *Limnognathia* including parthenogenesis (in part) [106,107] and widespread distribution [108,109]. Additionally, Syndermata is widely accepted as the sister phylum of Micrognathozoa [9–12]. In contrast to Micrognathozoa, *Brachionus*, as a model system, has been well studied, with its species supported by integrative taxonomy [110–113]. Additionally, the availability of transcriptomic data on this species complex makes it a suitable system to help us infer species limits in *Limnognathia*.

The two CLADES analyses produced conflicting results. The maximum likelihood classification based on the general model assigned the Greenland and Pyrenees populations as different species with moderate support (probability 0.713) (figure 3*a*). The general model has been shown to oversplit in some understudied taxa with complex speciation processes [25,28]. Classification using the custom model joined Greenland and Pyrenees with strong support (probability 0.8237) but split the Crozet population and the two Northern Hemisphere populations with moderate support (probability > 0.70) (figure 3*a*). We prefer the most conservative estimate across discovery and validation (PTP, VAE, custom CLADES) that clearly delimit only the Crozet population as a separate species. The Greenland and Pyrenees populations are likely undergoing incipient speciation.

### (f) COI divergence

Saturation tests showed that COI was not saturated in *Limnognathia* (electronic supplementary material, figure S14). Intrapopulation pairwise distances of the trimmed alignment were 0% in all three populations (electronic supplementary material, table S10). Delimiting species with COI barcoding is standard [114] although the use of fixed thresholds is contentious [115–117]. The distances between the Greenlandic and Pyrenees individuals (2.51%) are slightly higher than some well-studied taxa [118] and the common 2% divergence threshold for distinguishing species [119] but match values in species which are known to be overestimated by fixed thresholds [111,120–122]. The interpopulation divergences between Crozet and the other two populations (17.86%) are typical of congeneric species. Additionally, the difference between intra- and interpopulation divergence aligns with the 10 × threshold [123] for population versus species divergence among congeners [19,20].

### (g) Cryptic speciation in Micrognathozoa

Anatomical redundancy has been linked to morphological disparity, often leading to species diversity. Loss and gain of redundancy have led to evolutionary constraint and release from functional constraint, respectively [124–127]. With miniaturization, redundancy is often reduced [127,128]. Micrognathozoa, whether an example of extreme miniaturization or ancestrally small size, lack redundancy in certain structures such as muscles where each muscle may be formed by one cell [93]. The morphological conservatism between the two species of Micrognathozoa could stem from lack of redundancy shoehorning the phylum into morphological stasis.

Micrognathozoa’s presumed parthenogenetic reproduction may be linked to its morphological stasis and cryptic speciation. Asexual lineages with geographical isolation are expected to diverge and form distinct species [129,130], but their phenotypic differences may not be obviously detectable [131]. Cryptic species have been found in many asexual lineages [110,120,132–134]. In fact, cryptic speciation is more common and morphology more conserved in parthenogenetic rotifers compared with sexually reproducing relatives [129,135]. Micrognathozoans have high habitat specificity, often only found in certain moss clumps even within the same pools of water [16]. Extreme isolation from infrequent long-distance dispersal of micrognathozoans with parthenogenetic reproduction could produce divergent species with identical morphologies.

## 4. Conclusion

Through extensive morphological analyses using LM, SEM and CLSM [1–3], this study revealed no distinct differences between Northern Hemisphere and Crozet specimens. The deep phylogenetic divergence between these two groups*,* their extreme geographical separation, differences in COI and 18S rRNA sequences, and the results of extensive species delimitation analyses clearly suggest that there are at least two species of *Limnognathia*. The age of the divergences between Northern and Southern Hemisphere populations indicate long-distance dispersal potentially through high-elevation stepping stones, e.g. along the American Cordillera or directly from pole to pole. We highlight the ongoing search for more micrognathozoan populations globally in high latitudes and high elevations, particularly in South America, to fully understand the biogeographic history and dispersal capabilities of Micrognathozoa.

The novelty of this highly divergent lineage of Micrognathozoa on a Subantarctic island with broad implications for biogeography and speciation processes highlights the need for describing and naming this new species. Due to its indistinguishable morphology*,* we describe the new species using molecular data [136]. Highly conserved ribosomal RNA sequences have been used in the species descriptions of other understudied and morphologically conserved groups (e.g [19,137,138]) and are subsequently used here to establish fixed diagnostic characters.

## 5. Taxonomy

Phylum **Micrognathozoa** Kristensen and Funch, 2000

Family **Limnognathiidae** Kristensen and Funch, 2000

Genus ***Limnognathia*** Kristensen and Funch, 2000 figs. 1 and 2

urn:lsid:zoobank.org:act:B91157F7-BFB1−40B5-AC02-B658EE76F027

*Limnognathia* Kristensen and Funch, 2000:

*Type species: Limnognathia maerski* Kristensen and Funch, 2000

Amended diagnosis

Acoelomate, bilaterally symmetrical, unsegmented animals with intracellular plates in the non-ciliated dorsal and lateral epidermis. Epidermis is not syncytial. Adults 100−150 µm in length, juveniles ~85 µm. Body with three divisions: head, accordion-like thorax and abdomen. Transient dorsal anus on the abdomen. Females with one pair of ovaries, carrying a single large egg (40 × 60 µm) at a time. No males known, likely parthenogenetic.

Unique cuticular pharyngeal apparatus with multiple sclerotized elements organized into four main sets—a pair of large fibularia (including basal plates), central main jaws (including cauda and symphysis), ventral jaws (pseudophalangia and lamellae), dorsal jaws—and associated accessory sclerites. Ventral mouth opening with two anterior ciliary tufts. Paired pharyngeal ciliary tufts in the mouth cavity between the main jaws.

Ventral epidermis with unique arrangement of ciliophores. Head with anterior ciliary field, posterior ciliary field and three pairs of head ciliophores lateral to the mouth. Thorax and anterior abdomen with 15 midventral rows of 2−4 ciliophores forming a locomotory band. Posterior abdomen with cluster of 10 ciliophores forming an adhesive ciliary pad.

Sensory collar receptors formed by 1−3 cilia surrounded by microvilli arranged as minimum three apicalia, two pairs of frontalia, three to five pairs of lateralia, three pairs of dorsalia and two pairs of caudalia. Additional sensoria include two antero-lateral ‘brushes’ of shorter cilia (apical ciliary tufts) and a pair of long bendable cilia on the head (flagellar head structure). On the head, a pair of hyaline vesicles associated with a dense layer of microvilli possibly form phaosomal eyes.

Internal ciliary structures include two L-shaped oviducts and an anterior pair of protonephridia with four monociliated terminal cells, connected via intermediate ducts to a posterior pair of protonephridia with two terminal cells.

The central nervous system comprises a dorsal brain and four longitudinal ventral neurite bodies. The two main ventro-lateral and two ventro-median bundles are connected via subpharyngeal and posterior commissures. Pharyngeal ‘ganglion’ with paired ciliary receptors, potentially innervates the jaw musculature. Peripheral nerves extend ventrolaterally and dorsolaterally from the circumesophageal connectives. Body musculature consists of 7 pairs of longitudinal and 13 pairs of oblique dorso-ventral muscles.

***Limnognathia maerski*** Kristensen and Funch, 2000

urn:lsid:zoobank.org:act:F5DF3F6E-49F8-468D-95D9-A9A4342CBFD0

*Limnognathia maerski* Kristensen and Funch, 2000: 4−11, figs. 1 and 2

Material examined

*Molecular vouchers.* Greenland: Disko Island: four specimens collected in Isunngua Spring 69.716667, 51.933333, Aug.2013 (SRR29877518–SRR29877520); Spain: Parc Nacional d'Aigüestortes i Estany de Sant Maurici: three specimens collected in Bassa Nera 42.638183 , 0.924217 Jun.2022 (SRR29877515–SRR29877517).

Amended diagnosis

*L. maerski* is distinguished from *Limnognathia desmeti* sp. nov. by two fixed molecular diagnostic synapomorphies 18S rRNA (figure 3B). Coordinates refer to the position of the Sanger reference.

18S: 327(T), 780(G)

Remarks

Although reciprocally monophyletic, we currently cannot distinguish the Pyrenees population from Bassa Nera as a separate species from *L. maerski* based on the diagnostic characters here provided, which are requisite for erecting a new species These specimens share with *L. maerski* from Greenland the same molecular synapomorphies in 18S rRNA and are thus here tentatively classified as the same species. However, due to the results of most species delimitation analyses, the two populations are likely undergoing incipient speciation. Specimens from both Northern Hemisphere populations were frequently seen swimming with a characteristic slow, rotational upward-moving motion. Additional characters include two variable positions in 18S rRNA— (668(Y), 807(R)—that are homozygous in *L. desmeti* sp. nov. and 88 fixed molecular synapomorphies in COI that were consistent among all individuals sampled (COI: see electronic supplementary material, table S11 and figure S15). We tentatively include these as characters in remarks with the caveat of small sample size and acknowledge more variability might be uncovered in the future.

***Limnognathia desmeti*** Worsaae & Møller, sp. nov.

(figures 1b–d–2a–c, e–g)

urn:lsid:zoobank.org:act:7A9BCA8B-010B-44F0-9AE9-45044E037AF0

Material examined

*Holotype.* Crozet Archipelago: Île de la Possession: Pointe du Bougainville: NHMD-001801220, 1 specimen, −46.447919, 51.850842, 11.Dec.2019.

*Paratypes.* Crozet Archipelago: Île de la Possession: Pointe du Bougainville: NHMD-001801224, NHMD-001801225, NHMD-001801227, three specimens as permanent whole mounts on slides, −46.447919. 51.850842, 10.Dec.2019, NHMD-001801221, NHMD-001801222, NHMD-001801223, NHMD-001801226, four specimens as permanent whole mounts on slides, −46.447919, 51.850842, 11.Dec.2019

*Molecular vouchers.* Crozet Archipelago: Île de la Possession: four specimens collected at Pointe du Bougainville −46.446002, 51.849662, December 2019 (SRR29877511–SRR29877514).

Diagnosis

*L. desmeti* sp. nov. is distinguished from *L. maerski* by two fixed molecular diagnostic synapomorphies 18S rRNA (figure 3*b*). Coordinates refer to the position of the Sanger reference.

18S: 327(G), 780(T)

Type locality

Crozet Archipelago, Île de la Possession, Pointe du Bougainville, −46.447919, 51.850842, 130 m (electronic supplementary material, figure S16).

Etymology

This species is named in honour of Dr Willem H. De Smet, University of Antwerp, who first discovered *Limnognathia* on the Crozet Archipelago.

Remarks

This species is found in mosses from small freshwater creeks and ponds. Specimens were always observed attached to or gliding on the substrate and never swimming. Additional characters include three variable positions in 18S rRNA—1046(Y), 1344(M), 1684(Y)—that are homozygous in *L. maerski* and 88 fixed molecular synapomorphies in COI following the justification in the description for *L. maerski* (COI: see electronic supplementary material, table S11 and Figure 15)

## Data Availability

New sequences are deposited in the Sequence Read Archive (BioProject PRJNA1137119, Accession Numbers SRR29877511–SRR29877520). All matrices, alignments, CLADES files, custom scripts and tree files can be found on Harvard Dataverse [139]. All type specimens have been deposited in the Natural History Museum of Denmark. Supplementary material is available online [140].
